# Comparison of the CASA and InVEST models’ effects for estimating spatiotemporal differences in carbon storage of green spaces in megacities

**DOI:** 10.1038/s41598-024-55858-0

**Published:** 2024-03-05

**Authors:** Ruei-Yuan Wang, Xueying Mo, Hong Ji, Zhe Zhu, Yun-Shang Wang, Zhilin Bao, Taohui Li

**Affiliations:** 1 Department of Geographical Science, Guangdong University of Petrochemical Technology (GDUPT), Maoming, 525000 Guangdong Province China; 2 Graduate Institute, Fu Jen Catholic University, New Taipei City, 24205 Taiwan China; 3 Water Conservancy and Civil Engineering College, Xinjiang Agricultural University, Urumqi, 830052 Xinjiang China; 4 Key Laboratory of Plateau Geographic Processes and Environment Change of Yunnan Province, Faculty of Geography, Yunnan Normal University, Kunming, 650500 China

**Keywords:** Megacity, CASA model, InVEST model, Land use, Carbon storage of green space, Climate change, Forest ecology, Forestry

## Abstract

Urban green space is a direct way to improve the carbon sink capacity of urban ecosystems. The carbon storage assessment of megacity green spaces is of great significance to the service function of urban ecosystems and the management of urban carbon zoning in the future. Based on multi-period remote sensing image data, this paper used the CASA model and the InVEST model to analyze the spatio-temporal variation and driving mechanism of carbon storage in Shenzhen green space and discussed the applicability of the two models to the estimation of carbon storage in urban green space. The research results showed that, from 2008 to 2022, in addition to the rapid expansion of construction land, the area of green space and other land types in Shenzhen showed a significant decrease trend. The estimation results of the carbon storage model showed that the carbon storage of green space shows a significant trend of reduction from 2008 to 2022, and the reduction amounts are 0.8 × 106 t (CASA model) and 0.64 × 106 t (InVEST model), respectively. The evaluation results of the model show that, in megacities, the spatial applicability of InVEST model is lower than that of CASA model, and the CASA model is more accurate in estimating the carbon storage of urban green space. The research results can provide a scientific basis for the assessment of the carbon sink capacity of megacity ecosystems with the goal of "dual carbon".

## Introduction

Since the industrial revolution, with the acceleration of urbanization and the intensification of human activities, global warming has become one of the most important climate problems in the world^1^. Cities are the most frequent areas of human activity. Statistics show that more than 80% of global CO_2_ emissions come from urban areas, and in the near-surface area, the concentration of CO_2_ in the urban center is significantly higher than that in the urban periphery^2,3^. As the only direct carbon sink, urban green space plays an important role in maintaining carbon balance and ecosystem stability^4^. Nevertheless, the role of urban green space in the global carbon cycle has not been paid enough attention for a long time, and the estimation of carbon storage in terrestrial ecosystems is chiefly concentrated in natural areas such as forests, wetlands, and grasslands^5–7^. With the expansion of urban areas and the popularization of the concept of eco-city, the increase of urban green space has significantly improved the carbon sink capacity of urban ecosystems^8,9^. Therefore, the estimation of urban green space carbon storage is helpful to evaluate the carbon sink capacity of urban ecosystems.

In the early research on urban green space, scholars focused primarily on the theoretical demonstration of carbon sink capacity and the verification of carbon storage assessment methods^9,10^. Among them, Nowak and Crane^10^ were the first scholars to carry out quantitative analysis of carbon storage in urban green space, and the research results showed that urban green space can significantly regulate CO_2_ concentration in urban areas. Since then, the study of urban green space carbon storage has mainly adopted the sample site inventory method to establish the estimation model. However, this method requires field investigation and has a long time period, so it is not suitable for the study of large-scale space^11,12^. With the development of remote sensing technology, scholars can efficiently obtain long-time series data in a large area using that technology, which promotes the rapid development of large-scale regional research^13^. In recent years, scholars have begun to study the spatio-temporal characteristics and driving factors of urban green space carbon storage by using remote sensing models^14,15^. Studies have established that there are obvious differences in the carbon density of green space in different areas of the city, and its change is affected by the spatial distribution of green space, the intensity of urban development, and the structure of the plant community^15,16^. However, due to rapid urbanization and the lack of soil carbon pool data, there are significant differences in the estimation results of urban green space carbon storage by different remote sensing models (CASA and InVEST et al.), especially in megacities with rapid regional expansion^17,18^.

To sum up, the current research mainly uses a single remote sensing model to simulate and analyze, thus ignoring the differences between models. Although there are many models for predicting future carbon storage based on past carbon storage estimates (FLUS, CA, PLUS, etc.), the selection of the optimal model for estimating past urban carbon storage in the current study is still uncertain, which limits the accuracy of the results obtained in the combined study of prediction model and estimation model. Moreover, because CASA and InVEST models consider the influence of the mutual transformation between quantity, spatiotemporal distribution and LUCC, they simplify some structure construction mechanisms, break through the limitation of simulating nonlinear systems, and the simulation accuracy is high. In order to analyze the differences of remote sensing models and their applicability in megacities, we validate the estimates of CASA and InVEST models.

Shenzhen is one of the central cities of the Guangdong–Hong Kong–Macao Greater Bay Area, and it is also one of the four megacities in China. Statistics show that the urbanization rate of Shenzhen in 2021 has reached 99.81%^19^. Under the goal of “dual carbon”, it is very important for Shenzhen to manage carbon storage in urban areas^20^. Thus, the purpose of this paper is to clarify the evolution characteristics of urban green space carbon storage, which not only has important guiding significance for the future urban planning and urban carbon cycle research of Shenzhen, but also provides a new idea for the selection of carbon storage estimation model for megacities. This paper chooses this area as the research area, and its results of this study not only help to reveal the applicability of the two carbon storage models to the estimation of carbon storage of green space in megacities, but also have guiding significance for the assessment of carbon sink capacity in urban green space and urban spatial planning.

## Materials and methods

### Study area

**Figure 1:**
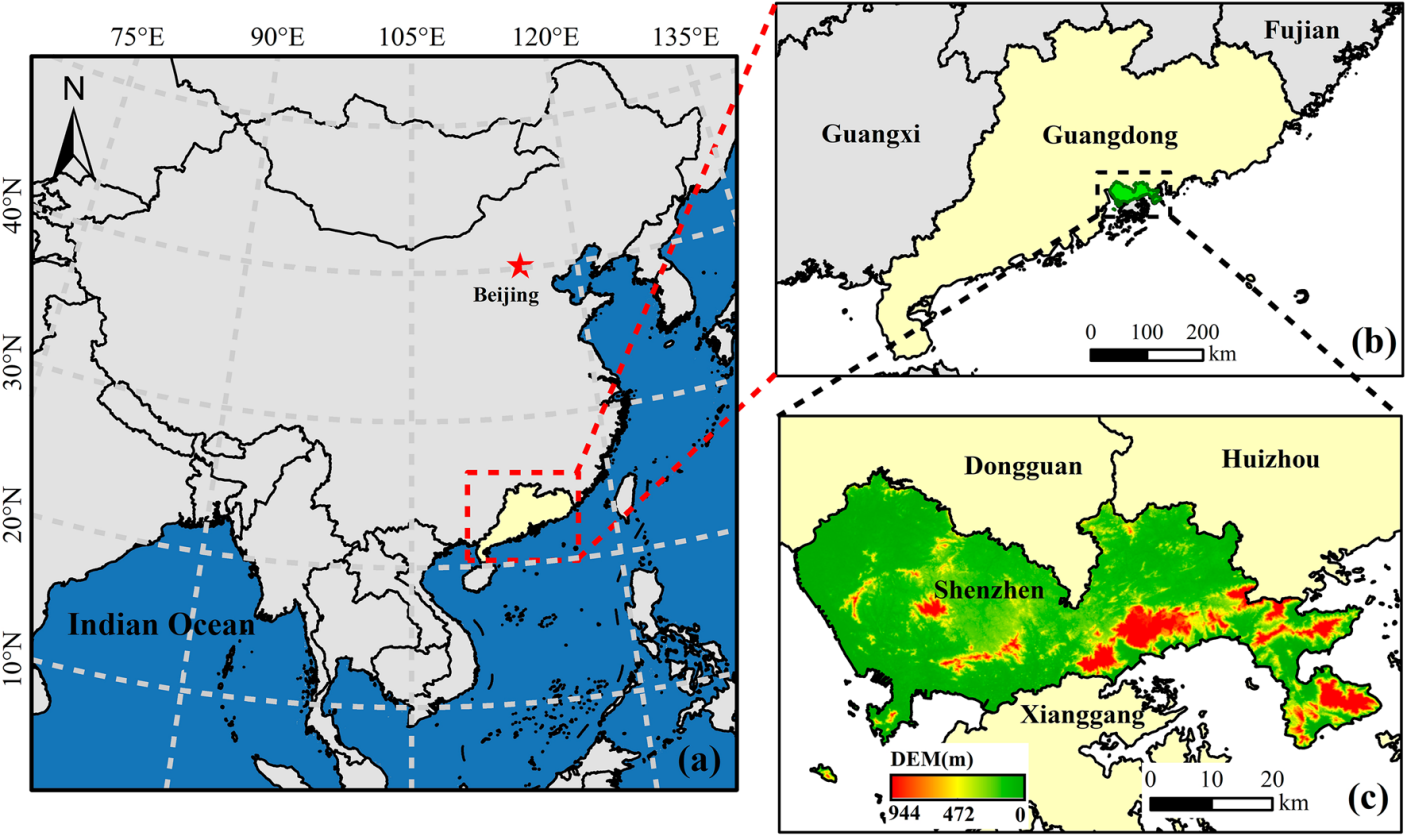
Location and terrain of the study area. (*Note*: The Arcgis10.8 software was used in this study. The data were obtained from The China Standard Map Service System. Drawing review No: GS(2020)4619.)

Located in the southern part of Guangdong Province (113° 43′–114° 38′ E, 22° 24′–22° 52′ N), Shenzhen is one of the four special economic zones in China (Fig. 1). That belongs to the subtropical monsoon climate zone; the average annual temperature is 22 °C, the annual rainfall is 1900 mm, and it is concentrated in April to September. In the past few decades, it has experienced a rapid urbanization process, from agricultural land and natural vegetation to construction land and artificial ecological land. Therefore, Shenzhen is a typical study area for the dynamic change in green space carbon storage in megacities^19,20^.

### Data sources and preprocessing

The remote sensing images used in this study are from the Geospatial Data Cloud [http://www.gscloud.cn (accessed on June 20, 2023)], and the dataset adopts remote sensing images of Landsat 5 and Landsat 8 (Table 1). The resolution of remote sensing images is 30 m, and the cloud coverage rate is less than 1%. Additionally, climate data are derived from the ERA5 monthly reanalysis dataset published by the European Center for Medium-Range Weather Forecasts [https://cds.climate.copernicus.eu/(accessed on June 30, 2023)], which includes rainfall, temperature, and solar radiation. And the forest carbon storage data are obtained from the National Tibetan Plateau Data Center [https://data.tpdc.ac.cn/ (accessed on August 30, 2023)].

**Table 1:** Data sources and description.

Year	Orbit number	The receiving time of the image	SENSOR_ID
2008	122/044	2008-12-01	TM
	121/044	2008-12-10	
2013	121/044	2013-10-05	OLI_TIRS
	122/044	2013-11-29	
2018	121/044	2018-03-09	OLI_TIRS
	122/044	2018-02-12	
2022	122/044	2022-12-24	OLI_TIRS
	121/044	2022-04-05	

The purpose of preprocessing remote sensing image data is to eliminate irrelevant information and extract useful real-life information to improve the reliability of supervised classification. Firstly, the ENVI 5.3 software is used to correct and clip remote sensing images. Secondly, the Normalized Difference Vegetation Index (NDVI) is extracted from the preprocessed images. Finally, the images are classified according to land use model and feature. Land use types are divided into cropland, green space, construction land, water areas, and unused land.

### Research methods

**Figure 2:**
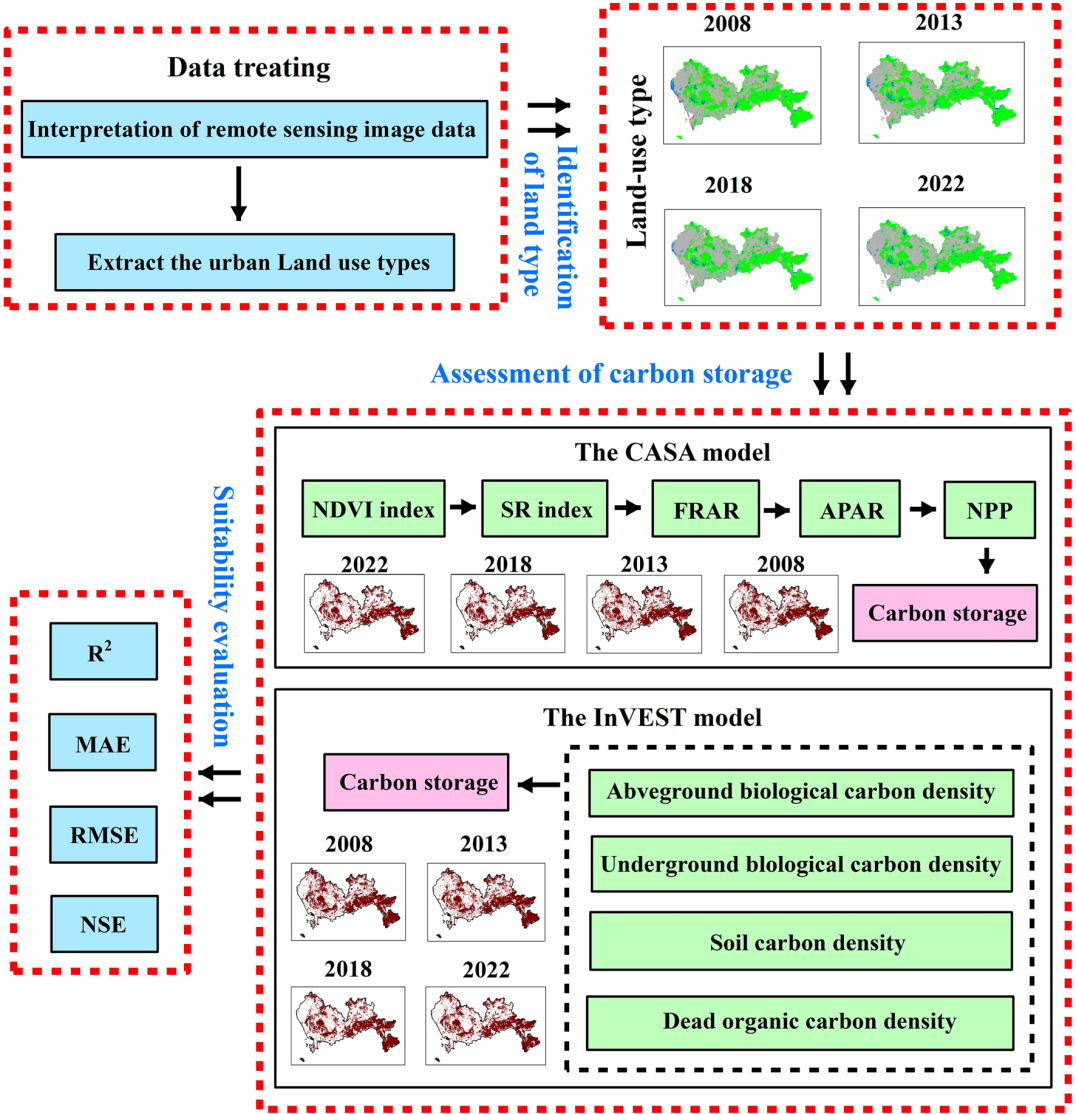
The framework of this study.

In this paper, the land use transfer matrix method is used to analyze the spatio-temporal evolution of land use in Shenzhen, and the CASA model and the InVEST model are used to estimate the carbon storage of green space. The research framework of this paper is shown in Fig. 2.

#### Land-use transfer matrix

The land use transfer matrix can describe the change in direction of different land use types in the region over different periods. Nowadays, it is also the most widely used method in land use research and can better reveal the spatio-temporal evolution process of land use patterns^21^. The calculation method is as follows:

1 $$ S_{ij} = \left[ {\begin{array}{*{20}l} {S_{11} } \hfill & {S_{12} } \hfill & \ldots \hfill & {S_{1n} } \hfill \\ {S_{21} } \hfill & {S_{22} } \hfill & \ldots \hfill & {S_{2n} } \hfill \\ \ldots \hfill & \ldots \hfill & \ldots \hfill & \ldots \hfill \\ {S_{n1} } \hfill & {S_{n2} } \hfill & \ldots \hfill & {S_{nn} } \hfill \\ \end{array} } \right] $$

where *S*_*ij*_ is the state of land use at the beginning and end of the study. *n* is the number of types of land use.

#### CASA model

The Carnegie-Ames-Stanford Approach (CASA) model is a typical light energy utilization model that is widely used because of its few parameters and simple calculation^22^. According to the results of land classification, this paper uses the CASA model to estimate the net primary productivity (NPP) of vegetation in Shenzhen. Finally, the carbon storage of green space was estimated by the carbon sequestration model of vegetation^23,24^. The calculation method is as follows:

1. The Fraction of Photosynthetically Active Radiation (FPAR):

In this paper, the maximum (*NDVI*_*max*_, *SR*_*max*_) and minimum (*NDVI*_*min*_, *SR*_*min*_) values of *NDVI* and *SR* of vegetation types were calculated by the cumulative frequencies of 95% (high vegetation coverage) and 5% (low vegetation coverage), respectively.

2 $$ {\text{FPAR}}_{{{\text{NDVI}}}} = \frac{{{\text{NDVI}}_{{\left( {{\text{x}},{\text{t}}} \right)}} - {\text{NDVI}}_{{\left( {{\text{i}},\min } \right)}} }}{{{\text{NDVI}}_{{\left( {{\text{i}},\max } \right)}} - {\text{NDVI}}_{{\left( {{\text{i}},\min } \right)}} }} \times \left( {{\text{FPAR}}_{\max } - {\text{FPAR}}_{\min } } \right) + {\text{FPAR}}_{\min } $$

3 $$ {\text{FPAR}}_{{{\text{SR}}}} = \frac{{{\text{SR}}_{{\left( {{\text{x}},{\text{t}}} \right)}} - {\text{SR}}_{{\left( {{\text{i}},\min } \right)}} }}{{{\text{SR}}_{{\left( {{\text{i}},\max } \right)}} - {\text{SR}}_{{\left( {{\text{i}},\min } \right)}} }} \times \left( {{\text{FPAR}}_{\max } - {\text{FPAR}}_{\min } } \right) + {\text{FPAR}}_{\min } $$

where *NDVI* is the Normalized Difference Vegetation Index. *NIR* is the reflection value of the near infrared band. *R* is the reflection value of the red light band.

Since NDVI values are also generated in non-vegetated areas, this paper calculates *FPAR* partitions according to the status quo of land classification. The calculation method is as follows:

4 $$ {\text{FPAR}}\left( {{\text{x}},{\text{t}}} \right) = \left\{ {\begin{array}{*{20}l} 0 \hfill & {{\text{Construction}}\;{\text{land}}} \hfill \\ {\upalpha {\text{FPAR}}_{{{\text{NDVI}}}} + \left( {1 + \upalpha } \right){\text{FPAR}}_{{{\text{SR}}}} } \hfill & {{\text{Green}}\;{\text{space}}} \hfill \\ \end{array} } \right. $$

where *FPAR* represents the photosynthetically active radiation absorbed by all vegetation within the pixel (*x*) over a period of time (*t*) (MJ·m^−2^·t^−1^). *α* is the adjustment coefficient (0.5) between the two methods.

(2) The Absorbed Photosynthetically Active Radiation (APAR):
  5 $$ {\text{APAR}}\left( {{\text{x}},{\text{t}}} \right) = {\text{SOL}}\left( {{\text{x}},{\text{t}}} \right) \times {\text{FPAR}}\left( {{\text{x}},{\text{t}}} \right) \times 0.5 $$
  where *APAR* represents the photosynthetically active radiation absorbed by all vegetation within the pixel (*x*) over a period of time (*t*) (MJ·m^−2^·t^−1^). *SOL* represents the total solar radiation received by the pixel (*x*) over a period of time (*t*) (MJ·m^−2^·t^−1^).
(3) The Net Primary Productivity (NPP) of vegetation:
  6 $$ {\text{NPP}}\left( {{\text{x}},{\text{t}}} \right) = {\text{APAR}}\left( {{\text{x}},{\text{t}}} \right) \times\upvarepsilon \left( {{\text{x}},{\text{t}}} \right) $$
  where is the actual light energy utilization rate of vegetation in pixel (*x*) over a period of time (*t*) (g C MJ^−1^). *NPP*_(*x, t*)_ is the NPP of the vegetation within the pixel (*x*) within a period of time (t) (g C m^2^).
(4) The Carbon Storage of Green Space (GSGP):

Based on the calculation of NPP with CASA model, this study uses the carbon sequestration model of vegetation to estimate the carbon storage of green space in the study area^25^.

7 $$ {\text{E}} = {\text{T}} \times\updelta \times C $$

where *E* is the carbon sequestration amount of land cover type (*t*); *T* is the land area corresponding to the land cover type (hm^2^); *δ* is the NPP of land cover type (g C·m^2^); *C* is the conversion factor between vegetation biomass and carbon content (0.45).

#### InVEST model

The InVEST model’s carbon storage module categorizes ecosystem carbon storage into four categories: aboveground carbon storage, underground carbon storage, soil organic carbon storage, and dead organic matter carbon storage. According to land use classification, the average carbon density of four basic types of different land classes was calculated to obtain the total carbon storage in the study area^26,27^. The specific calculation formula is:

8 $$ C_{total} = \left( {C_{above} + C_{below} + C_{soil} + C_{dead} } \right) \times A_{i} $$

where *i* is the average carbon density of the earth class, *A*_*i*_ is the area of the earth class, and *C*_*toal*_ is the total carbon storage of all land types (t·hm^−2^), *C*_*above*_ is the aboveground carbon storage (t·hm^−2^), *C*_*below*_ is the underground carbon storage (t·hm^−2^), *C*_*soil*_ is the soil organic carbon storage (t·hm^−2^), *C*_*dead*_ is the dead organic matter carbon storage (t·hm^−2^).

According to the needs of the research and the requirements of the model, the carbon density data of different land classes in this paper came from the National Ecological Science Data Center [http://www.cnern.org.cn/ (accessed on June 25, 2023)], and were combined with the research results of Lin et al.^28^. The carbon density data of different land classes in Shenzhen was calibrated (Table 2).

**Table 2:** Carbon density values of various land use types (Unit: t·hm^−2^).

Land use type	Aboveground carbon storage	Belowground carbon storage	Soil organic carbon storage	Dead organic matter carbon storage
Cropland	16.56	3.31	10.84	0
Green space	20.57	6.17	22.57	4.71
Water area	0.01	0	0	0
Construction land	8.69	1.74	15.88	0
Unused land	13.55	2.71	0.83	0

#### Evaluation indicators of the model

In this study, the coefficient of determination (*R*^2^), Mean Absolute Deviation (*MAE*), Root Mean Squared Error (*RMSE*) and Nash–Sutcliffe efficiency coefficient (*NSE*) were used to evaluate the estimates of the CASA model and the InVEST model^29,30^. The specific calculation formula is:

9 $$ R^{2} = \left( {\frac{{\sum\nolimits_{i = 1}^{N} {\left( {X_{i} - \overline{X}} \right)\left( {Y_{i} - \overline{Y}} \right)} }}{{\sqrt {\sum\nolimits_{i = 1}^{N} {\left( {X_{i} - \overline{X}^{2} } \right)} } \sqrt {\sum\nolimits_{i = 1}^{N} {\left( {Y_{i} - \overline{Y}^{2} } \right)} } }}} \right)^{2} $$

10 $$ {\text{MAE}} = \frac{{\sum\nolimits_{i = 1}^{N} {\left| {X_{i} - Y_{i} } \right|} }}{N} $$

11 $$ RMSE = \sqrt {\sum\nolimits_{i = 2}^{N} {\frac{1}{N}\left( {X_{i} - Y_{i} } \right)^{2} } } $$

12 $$ NSE = 1 - \frac{{\sum\nolimits_{i = 1}^{N} {\left( {X_{i} - Y_{i} } \right)^{2} } }}{{\sum\nolimits_{i = 1}^{N} {\left( {X_{i} - \overline{X}} \right)^{2} } }} $$

where *X*_*i*_ is the measured value of i the time series, *Y*_*i*_ is the simulated value of i the time series, *N* is the number of samples, i is the time series.

## Results

### Spatio-temporal characteristics of land use

**Figure 3:**
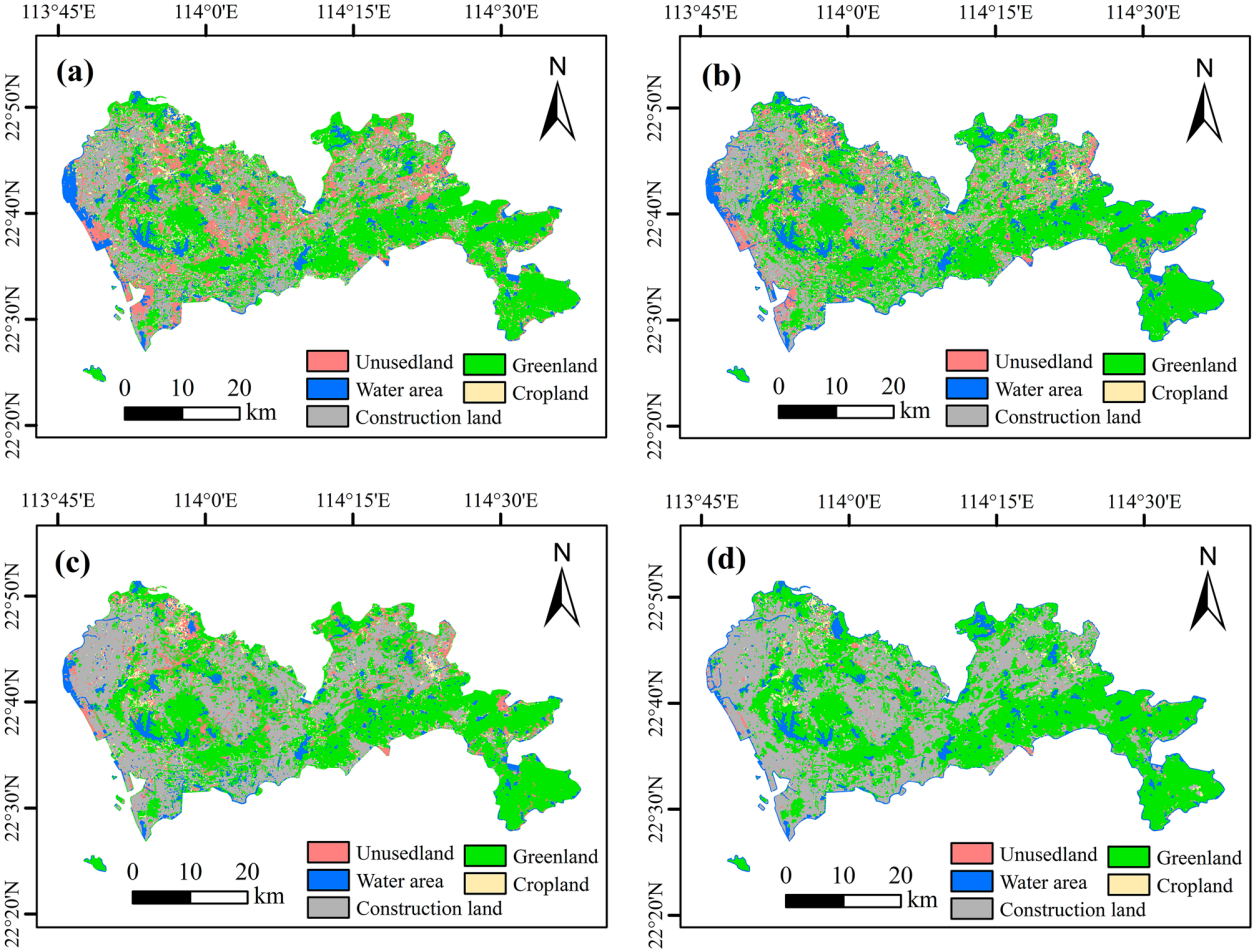
Land use distribution map of Shenzhen from 2008 to 2022 (**a**–**d** shows 2008, 2013, 2018, and 2022).

This paper uses the support vector machines (SVM) of the supervised classification method to obtain land use data for Shenzhen in four periods, then carries out classified statistics (Table 3), and characterizes the spatial distribution map of land use in multiple periods (Fig. 3). The results showed that the land use in Shenzhen was mainly construction land and green space (accounting for more than 90% of the total area), and the proportion of water area, unused land, and cropland was relatively low. In terms of spatial distribution, the eastern region is dominated by green space (high vegetation coverage rate), while the western region is dominated by construction land (high population density).

**Table 3:** Area changes of different land types from 1990 to 2020 (Unit: km^2^).

Land use type	2008		2013		2018		2022	
	Area	Percentage (%)	Area	Percentage (%)	Area	Percentage (%)	Area	Percentage (%)
Cropland	2161	1.1	3384.9	1.7	2003.0	1.03	1271.0	0.7
Green space	91677.6	46.9	89531.3	45.8	79990.0	40.94	79640.9	41
Water area	6997.5	3.6	6925.3	3.5	5832.6	2.99	6187.5	3.2
Construction land	85490.7	43.8	89056.0	45.6	102989.5	52.27	106957.2	55
Unused land	9039.1	4.6	6468.4	3.3	4549.0	2.33	1309.1	0.7

The results show that from 2008 to 2022, the area of construction land in Shenzhen presents a significant upward trend (+ 11.2%), in which the area of construction land increases by 21466.5 hm^2^. This phenomenon is related to the sea reclamation project implemented in Shenzhen^31^. Among them, Baoan District and Nanshan District are the main concentrations of sea reclamation. Due to the adjustment of Shenzhen’s industrial structure and economic model, the cropland showed an increasing trend (+ 0.6%) from 2008 to 2013 (+ 1223.9 hm^2^), and then showed a decreasing trend (− 1%) from 2013 to 2022 (− 2113.9 hm^2^). The area of green space (− 5.9%), water area (− 0.4%), and unused land (− 3.9%) showed a significant downward trend from 2008 to 2022, indicating that with the development of urbanization, Shenzhen’s land demand increased, and green space, unused land, and water area were developed into construction land.

**Figure 4:**
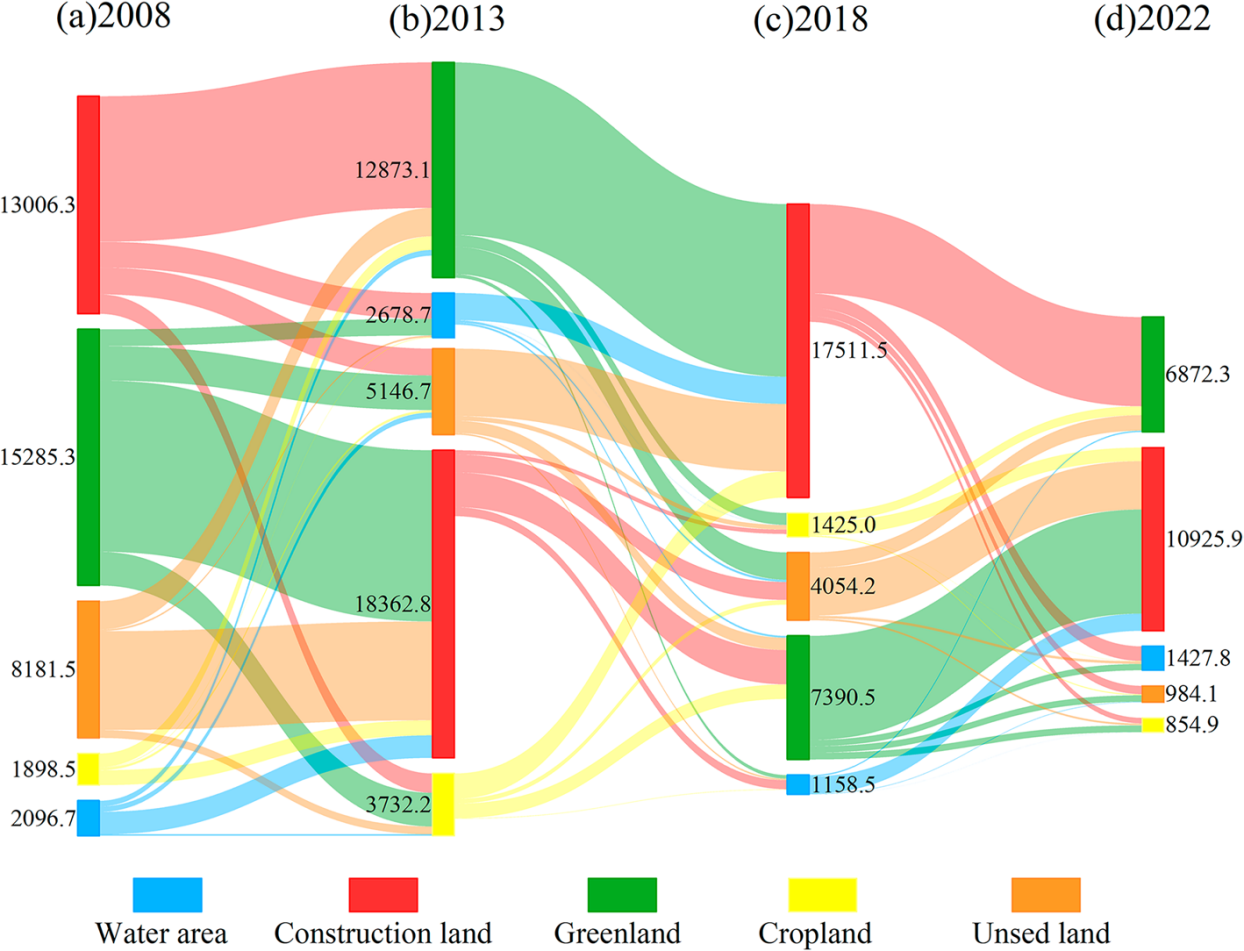
Land use transfer in Shenzhen from 2008 to 2022.

In order to understand the change direction of different land types and the evolution process of regional land types, this paper conducted a quantitative analysis of the transfer types of land use in Shenzhen over multiple periods. The results show that (Fig. 4): (1) In 2008–2013, the retention rate of construction land was relatively high. Green space is the main type of land transfer, and the area of green space transformed into construction land accounts for 67.8% of the total area transferred out. The area of cropland converted into construction land (897.1 hm^2^), and the area converted into green space (815.4 hm^2^). The unused land and water area were converted into construction land. (2) In 2013–2018, construction land and green space were the main types of land transfers. The cropland was transformed into construction land and green space. The water area and unused land were converted into construction land. (3) In 2018–2022, there is a phenomenon of mutual conversion between green space and construction land. The water areas were converted into construction land. The unused lands were converted into construction land (2861 hm^2^). The cropland is converted to construction land and forest land.

Overall, in 2008–2022, except for the construction land, other land use types showed a trend of decrease. Especially after 2013, construction land showed a significant growth rate. Cropland, green space, and unused land are the main sources of construction land. The phenomenon was due to the increasing demand for construction land in the process of urbanization. Among them, construction land is one of the main factors affecting carbon emissions, and the increase in construction land will lead to an increase in urban carbon emissions. Meanwhile, as the main carbon sink of urban ecosystems, the reduction of vegetation in green spaces will significantly reduce the carbon storage of urban ecosystems and the carbon sequestration capacity of soil.

### Estimation of carbon storage of the green space by CASA model

Based on the CASA model and the carbon sequestration equation of vegetation, the carbon storage of Shenzhen’s green space was calculated. The results show that (Table 4): In 2008–2022, the carbon storage of green space showed a trend of fluctuation reduction (0.8 × 10^6^ t). Among them, the highest value of carbon storage in Shenzhen's green space appeared in 2008 (3.31 × 10^6^ t), and the lowest value appeared in 2022 (2.51 × 10^6^ t). Combined with relevant studies^32^, the expansion of cities and the reduction of green space are the main reasons for the decline of green space carbon storage. In addition, climate effects such as global warming and urban heat island may lead to inter-annual changes in regional rainfall, temperature, solar radiation, and other climate factors, which in turn affect the seasonal growth and development of vegetation, resulting in inter-annual changes in carbon storage in green space^26,33^.

**Table 4:** Statistical of carbon storage and green space area from 2008 to 2022 (CACS model).

Year	2008	2013	2018	2022
Area (hm^2^)	9.17 × 10^4^	8.95 × 10^4^	7.99 × 10^4^	7.96 × 10^4^
Carbon storage of the green space (t)	3.31 × 10^6^	2.91 × 10^6^	3.11 × 10^6^	2.51 × 10^6^

**Figure 5:**
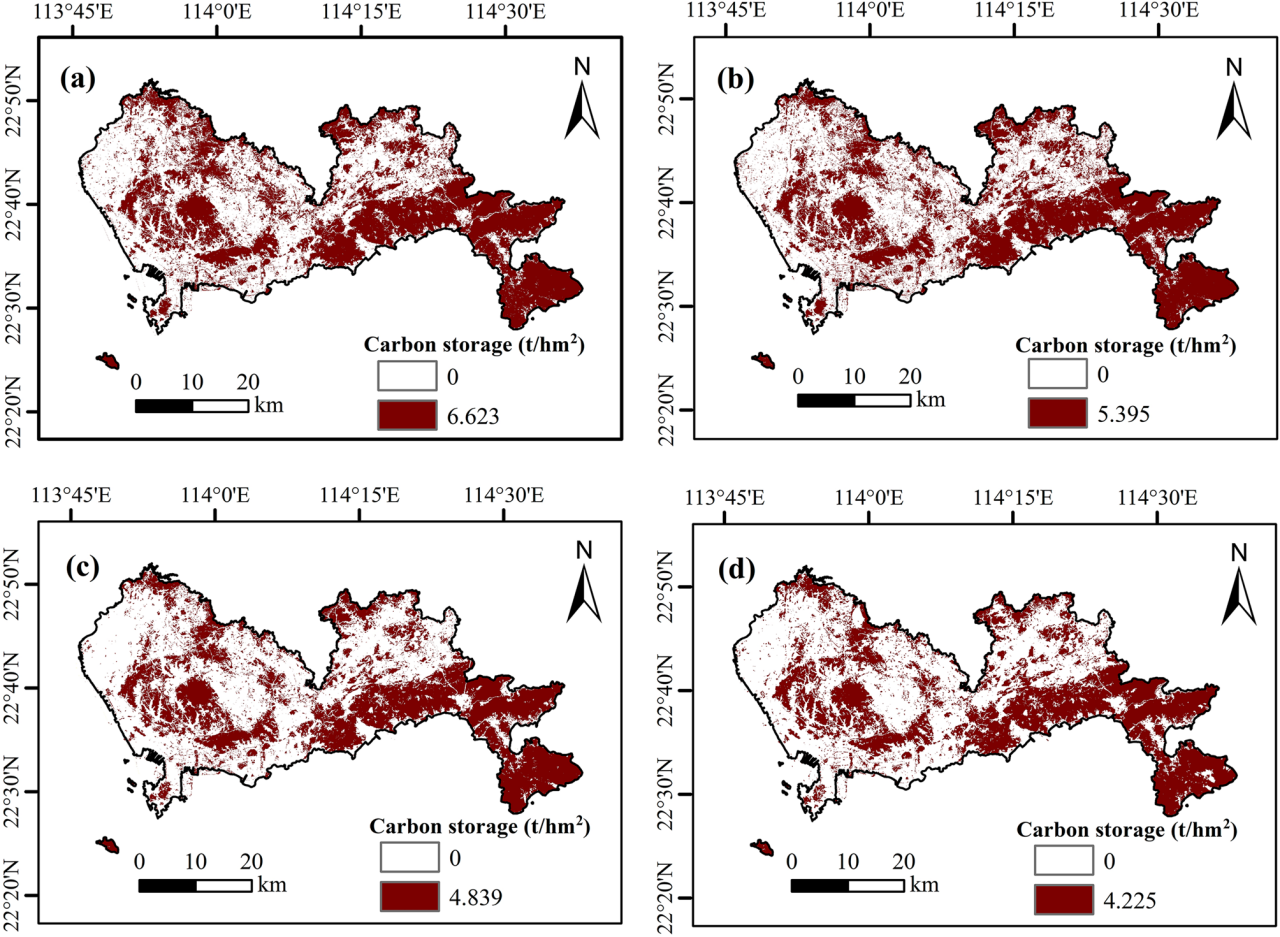
Carbon storage distribution map of green space in Shenzhen from 2008 to 2022 based on CASA model (Figure a-d shows 2008, 2013, 2018, and 2022).

The spatial distribution of carbon storage in Shenzhen's green space (Fig. 5) showed that the spatial distribution of carbon storage in green space during 2008–2022 was basically the same, with the overall distribution characteristics of “low in the west and high in the east” (The distribution range of carbon storage in green space is 31.53 to 38.92 t hm^−2^). The regions with high carbon storage value are mainly distributed in the mountainous areas in the east of Shenzhen (such as Dapeng District and Yantian District), which have strong carbon storage capacity due to their large slope and high vegetation coverage^20^. However, the regions with low carbon storage values are distributed in the western and central areas of Shenzhen (such as Baoan District and Pingshan District, etc.), which have weak carbon storage capacity due to their lower altitude and higher urbanization level^19^.

### Estimation of carbon storage of the green space by InVEST model

**Figure 6:**
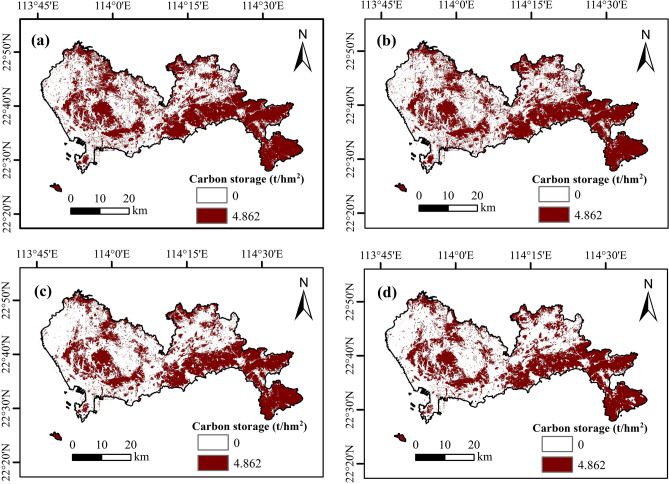
Carbon storage distribution map of green space in Shenzhen from 2008 to 2022 based on InVEST model (Figure a-d shows 2008, 2013, 2018, and 2022 respectively).

In order to estimate the carbon storage of Shenzhen’s green space (Table 5), this paper first calculates the total carbon storage (7.42 × 10^6^ to 7.17 × 10^6^ t) during 2008–2022 through the carbon module of the InVEST model. Secondly, this paper used ArcGIS 10.8 software to statistically partition the carbon storage of green space. Finally, according to the results, we presented the spatial distribution of carbon storage in Shenzhen green space (Fig. 6). The results showed that the carbon storage in Shenzhen's green space decreased from 4.95 × 10^6^ to 4.84 × 10^6^ t (2008–2013), decreased from 4.84 × 10^6^ to 4.32 × 10^6^ t (2013–2018), and decreased from 4.32 × 10^6^ to 4.31 × 10^6^ t (2018–2022). In general, the change trend of green space carbon storage is consistent with that of the green space area. As the InVEST model mainly estimated carbon storage by land type area, the covered area of green space determined the carbon storage in the region.

**Table 5:** Statistical of carbon storage from 2008 to 2022 (InVEST model).

Year	2008	2013	2018	2022
Carbon storage of the green space (t)	4.95 × 10^6^	4.84 × 10^6^	4.32 × 10^6^	4.31 × 10^6^
Total carbon storage (t)	7.42 × 10^6^	7.40 × 10^6^	7.17 × 10^6^	7.18 × 10^6^

The spatial distribution of carbon storage in Shenzhen's green space (Fig. 6) showed that the spatial distribution of carbon storage based on the InVEST model is consistent with the CASA model, with the overall distribution characteristics of “low in the west and high in the east” (The distribution range of carbon storage in green space is 53.98 to 54.15 t hm^−2^). This result is related to the spatial distribution of green space in Shenzhen. Among them, the carbon storage of Shenzhen’s green space accounts for more than 60% of the total carbon storage. Combined with the data in Table 3, it is shown that the decline rate of total carbon storage from 2008 to 2022 is lower than that of the carbon storage of green space (Table 5) because the decline rate of water area, cropland and unused land is lower than that of green space. By comparing the results in Tables 4 and 5, it can be seen that the estimated result on the InVEST model is significantly higher than that of the CASA model.

### Comparison of the InVEST model and the CASA model

In this paper, two estimation models (CASA model and InVEST model) were used to estimate the carbon storage of Shenzhen's green space, respectively. The spatial distribution of carbon storage showed consistent changes in the two models (Figs. 5, 6), that is, the overall spatial pattern was “low in the west and high in the east”. This result is related to the spatial distribution of green space in Shenzhen. Therefore, in order to analyze the applicability of different models in urban green space carbon storage estimation, this paper adopts different index methods to analyze the estimation results of different models. Because the observed data cannot be analyzed at a spatial scale, we used a dataset for forest carbon stocks (published in February 2023). Although this dataset cannot cover all vegetation types, it does include spatial trends of forest carbon stocks over different time series and the accuracy of this data set can meet the needs of current research^34^. In summary, we use this data set as the true value to verify the spatial accuracy of different models. The results in Table 6 show that, overall, In the CASA model, R^2^, MAE, RMSE and NSE are superior to the INVEST model. This shows that the CASA model has better spatial applicability than the InVEST model.

**Table 6:** Accuracy verification of model results.

Model	R^2^	MAE	RMSE	NSE
CASA	0.997	0.123	0.162	0.989
InVEST	0.982	0.251	0.273	0.978

Although both the CASA model and the InVEST model estimate the carbon storage of green space based on the area of green space, the estimation results of the CASA model are significantly lower than the InVEST model (Tables 4, 5). This is because there are significant differences in the thickness of vegetation in different months, which leads to differences in NDVI and SR index in the region, resulting in lower carbon storage results calculated by the CASA model combined with the carbon sequestration model^35,36^. According to existing research results, there are certain differences in carbon sink capacity among different vegetation types (Table 7)^37,38^. Therefore, although the InVEST model is a mature carbon storage estimation model, there is high uncertainty in the assessment of the carbon storage of a single land class, especially in urban areas with rapid land circulation. The results of the carbon storage of urban green space estimated by the CASA model combined with the carbon sequestration model may be better than the InVEST model.

**Table 7:** Carbon density of different green space types in Shenzhen (Mg C/hm^2^).

Types of the green space	2005 year	2010 year	Change rate (%)	Average value
Arborous layer	21.62	24.42	12.95	23.02
Shrub layer	4.18	4.12	-1.44	4.15
Herb layer	0.54	0.5	-7.41	0.52
Litter layer	2.98	3.13	5.03	3.06
Average value	7.33	8.04	9.69	7.69

## Discussion

### Driving factors of carbon storage of in urban green space

**Figure 7:**
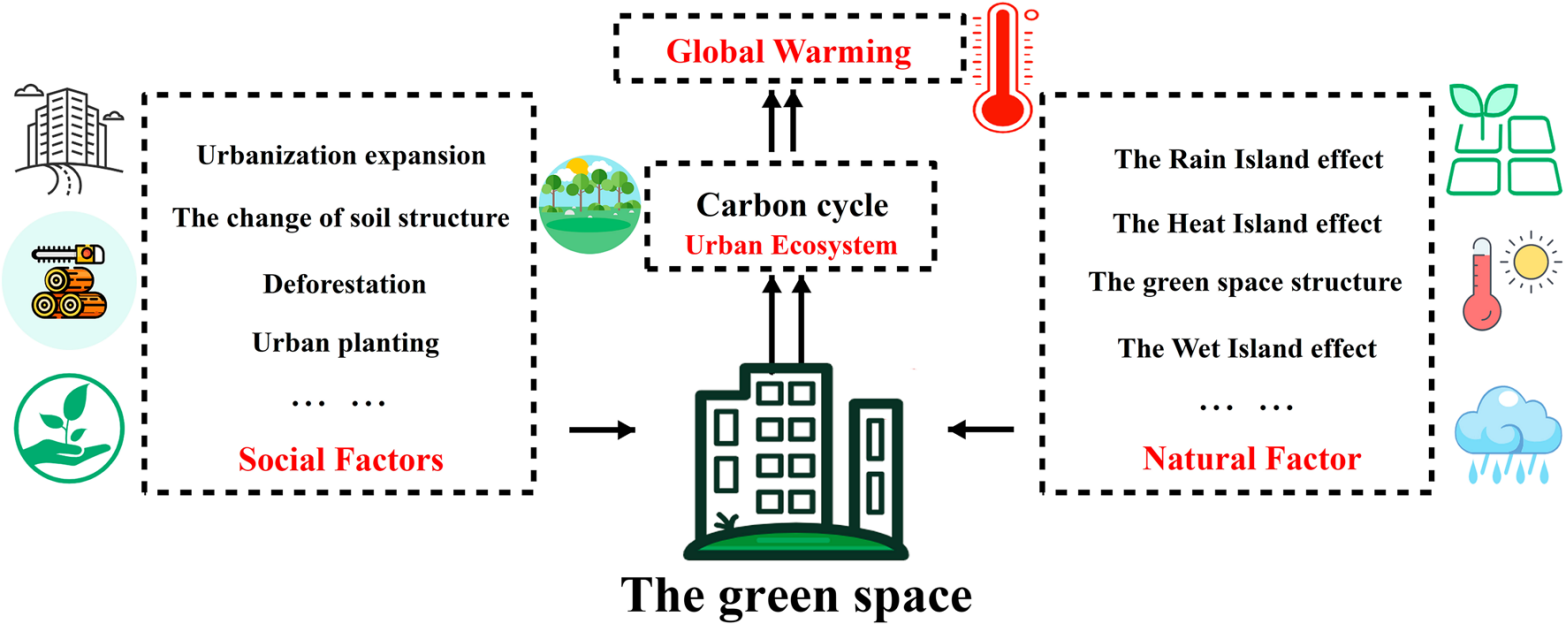
Driving mechanism of the carbon storage in urban green space.

Urban green space is one of the important components of carbon sink function in urban ecosystems^37^. According to the conclusion in “Comparison of the InVEST model and the CASA model” section and previous studies^39–43^, the carbon storage of urban green space is jointly affected by social factors and natural factors (Fig. 7). From the perspective of social factors, the encroachment of construction land on green space, the change of soil structure, deforestation, and urban planting will significantly change the carbon storage of urban green space in a short period of time, resulting in a significant decrease in the carbon storage of urban green space on a long-term scale and a fluctuating trend on a short-term scale^39–41^. From the perspective of natural factors, the change in urban climate caused by the rain island effect, the maintenance of the optimal temperature of vegetation by the heat island effect, and the enrichment of urban green space structure also affect the growth and development of vegetation to a certain extent and thus lead to the change in carbon storage of urban green space^42,43^. In contrast, under similar climatic conditions, the carbon storage of urban green space may be influenced more by human activities.

### Limitations and future studies

The current problem is that it is difficult to obtain long time series and large space measured data, which hinders the development of models and the accuracy of estimation results^17,18^. At the same time, without accurate assessment of urban green space carbon storage, it will be difficult for us to accurately formulate urban carbon management policies. In addition, urban green space differ significantly from natural green space, and urban green space has a high degree of fragmentation. In contrast, the natural green space with concentrated spatial distribution has a relatively high exchange rate of matter and energy^46^. Therefore, we believe that the spatial fragmentation of urban green space can be reduced through reasonable urban planning and management measures, so as to improve the carbon sink capacity of urban green space.

In summary, as the region with the fastest urbanization process, the transfer rate of land use type in megacity is significantly higher than that of other urban areas in a short time, in which makes it more difficult to estimate the carbon storage of urban green space^44,45^. Meanwhile, as the most important component area of urban ecosystems, the carbon storage assessment of megacity green spaces is of great significance to the service function of urban ecosystems and the management of urban carbon zoning in the future^46,47^. However, due to the regional urbanization level and the inconsistency of impact indicators, the driving factors of urban green space carbon storage cannot be quantified. This is one of the problems that needs to be solved in the future.

## Conclusion

This study first interpreted the land use type of Shenzhen from 2008 to 2022 through remote sensing image data, then estimated the carbon storage of Shenzhen's green space using the CASA model and the InVEST model, respectively, and finally discussed the driving factors of carbon storage in urban green space. The following three conclusions can be drawn:

1. From 2008 to 2022, the area of urban green space, unused land, water area, and cropland in Shenzhen showed a significant decrease trend, while the construction land showed a rapid expansion trend to the surrounding areas. Among them, the land type in the eastern region is mainly urban green space, and the land type in the western region is construction land.
2. In terms of temporal, the carbon storage of green space in Shenzhen showed a significant decrease trend from 2008 to 2022, and the reduction of carbon storage was 0.8 × 10^6^ t (the CASA model) and 0.64 × 10^6^ t (the InVEST model), respectively. Among them, the estimated result by the CASA model is lower than that of the InVEST model.
3. In terms of spatial distribution, the spatial distribution of carbon storage in Shenzhen's green space by the CASA model and the InVEST model is quite consistent, showing a spatial pattern of “low in the west and high in the east”. The high-value area of carbon storage is distributed in the mountainous area in the east of Shenzhen, and the low-value area of carbon storage is distributed in the plain area in the west and middle.
4. The evaluation results of the model show that in the CASA model, R^2^, MAE, RMSE and NSE are superior to the INVEST model. The CASA model has better spatial applicability than the InVEST model.

## Data Availability

The data is available from the corresponding authors upon request.
